# A Novel Solid Artificial Diet for *Zeugodacus cucurbitae* (Diptera: Tephritidae) Larvae With Fitness Parameters Assessed by Two-Sex Life Table

**DOI:** 10.1093/jisesa/ieaa058

**Published:** 2020-08-18

**Authors:** Xiangrui Liu, Xianwu Lin, Jing Li, Fen Li, Fengqin Cao, Rihui Yan

**Affiliations:** 1 Key Laboratory of Green Prevention and Control of Tropical Plant Diseases and Pests (Hainan University), Ministry of Education, Haikou, Hainan Province, China; 2 College of Ecology and Environment, Hainan University, Haikou, Hainan Province, China; 3 College of Plant Protection, Hainan University, Haikou, Hainan Province, China

**Keywords:** *Zeugodacus cucurbitae*, melon fly, artificial diet, fitness parameters, two-sex life table

## Abstract

The melon fly, *Zeugodacus cucurbitae* (Coquillett), is a serious pest of many fruits and vegetables throughout the world. Here we have developed an easy and quick-to-prepare solid medium with multiple benefits including reductions in post-rearing waste, storage space, and labor for rearing *Z. cucurbitae* larvae. The development time from egg to pupa was 19.11 d when larvae were reared on the artificial diet, slightly longer than 17.73 d on pumpkin and 17.13 d on cucumber. *Zeugodacus cucurbitae* achieved higher values of pupal weight, length, and width on the artificial diet than two natural diet controls. The rates of pupation and adult emergence of *Z. cucurbitae* grown on the solid medium were comparable with those on pumpkin and cucumber. Furthermore, determined by age-specific two-sex life table method, the age-specific survival rate of *Z. cucurbitae* was higher on the artificial diet than cucumber but lower than pumpkin. The reproductive ability and population dynamics of *Z. cucurbitae* were not significantly affected on the solid medium compared with those on the two natural diets. The results suggest that our solid artificial diet is excellent for rearing *Z. cucurbitae* larvae in laboratory and may be used for its mass rearing, therefore facilitating its research and control.

The melon fly, *Zeugodacus cucurbitae* (Coquillett), previously classified as *Bactrocera cucurbitae* (Virgilio et al. 2015), is widely distributed in temperate, subtropical and tropical regions of the world. Melon fly damages over 80 different host plants, and represents a very severe economic pest of cucurbitaceous vegetables, particularly in South China and South Asia. Females lay eggs on hosts and the maggots bore into the pulp tissue, consequently making fruits rotten or in low quality. The damages from melon fly may cause losses of 30–100% production, depending on different seasons and host species (Dhillon et al. 2005, Luo et al. 2017). As *Z. cucurbitae* larvae largely feed inside fruits, they have long been notoriously difficult to be controlled by insecticides that are commonly used for controlling many pests and diseases. Thus, there is a need to carry out studies on basic insect biology and to develop new management strategies for better controlling it, for example, genetic control and sterile insect technique (SIT) (Du et al. 2019, Kittayapong et al. 2019). SIT acts by releasing a large number of sterile males that can compete with wild-type males to mate with females into the environment, making the females in the natural population to produce no offspring and thereby decreasing the target population (Klassen and Curtis 2005, Thistlewood and Judd 2019).

In either laboratory research or practical pest control, it is essential to have a culturing medium capable of laboratory and mass rearing of *Z. cucurbitae*. Artificial diets are usually used for rearing a large number of insects as it is not convenient to obtain natural host plants throughout the entire year. Natural food may contain harmful materials, such as insecticides and pathogens, therefore influencing precision and accuracy of research results. While various artificial diets have been successfully developed for rearing economically important fruit fly pests, for example, *Bactrocera dorsalis* (Jayanthi and Verghese 2002, Liu et al. 2015), *Bactrocera zonata* (Rabab et al. 2016), and Mediterranean fruit fly (Tanaka et al. 1969, Vargas and Carey 1989), only a few artificial diets have been examined or developed for *Z. cucurbitae* (Tanaka et al. 1969, Chang et al. 2004, Huang and Chi 2012, Jaleel et al. 2018).

The wheat-bran-based solid diet has long been used worldwide for mass rearing of tephritid fruit flies including *Z. cucurbitae*. Although this diet provides good nutrition to the development of *Z. cucurbitae* larvae, it has some disadvantages such as high cost, intensive labor, difficult management of used diets and containers, and potential contamination from pesticides and other chemicals and pathogens (Tanaka et al. 1969).

To overcome the aforementioned problems, a liquid diet and rearing system primarily using sponge cloth has been developed (Chang et al. 2004). However, it still has some problems. In this system early larvae can readily drop into the liquid so that it needs special care. Pupal recovery is significantly lower from the liquid diet compared to that from the diet developed by Tanaka et al. (1969). In addition, sponge cloth cleaning and wash are still required and therefore the effect of labor saving is reduced to some extent. A semiartificial diet that contains agar and banana has been examined for rearing four *Bactrocera* species including *Z. cucurbitae*; however, it is only suitable for *B. dorsalis* (Jaleel et al. 2018).

The age-stage, two-sex life table has been applied to analyze population ecology and dynamics as it has multiple advantages compared to the traditional life table (Jaleel et al. 2018, Liang et al. 2019, Tang et al. 2019). Here, we use age-stage, two-sex life table to evaluate the growth and reproduction of the populations reared on cucumber, pumpkin, and our solid medium that we have developed for *Z. cucurbitae* larvae. The assessment results suggest that our artificial diet is suitable for the larval development of *Z. cucurbitae* in laboratory and might be used for its mass rearing in the application of SIT and genetic pest control.

## Materials and Methods

### Insects and Rearing

The melon flies used in this study were collected from City West campus of Hainan University and maintained in an environment-controlled room set at 26 ± 1°C, 65 ± 5% RH, and a photoperiod of 14:10 (L:D) h. Before our experiments, *Z. cucurbitae* had been reared for at least two generations with larvae on three diets: pumpkin, cucumber, and our artificial diet in petri dishes (9 cm × 1.5 cm) for acclimatization to each diet. Adult flies were reared in cages (35 cm × 35 cm × 35 cm), inside which contained water-soaked cotton wool in a plastic bowl (11.5 cm diameter × 7 cm height) and yeast extract (Oxoid, Basingstoke, Hants, UK) and sugar (1:1) in a petri dish (9 cm × 1.5 cm).

### Diet Preparation

The artificial diet is composed of corn meal, soy flour, brewer’s yeast (Instant dry yeast, Angel, Yichang, China), yeast extract, granulated sugar, glucose, CaCl_2_·2H_2_O, agar, small slices of pumpkin that were softened by boiling in water, water, and propionic acid (Table 1). The ingredients were mixed in an electric cooker (CFXB15-5M, Guangdong Hemispherical Industrial Group Co., Ltd., Zhanjiang, China) and 10,000 ml of water was added. The mixture was then heated with frequent agitation until the powder was completely dissolved. After cooling to 60°C, 48 ml of propionic acid was added, and the mixture was aliquoted into petri dishes or other containers with a peristaltic pump (YZ1515x, Baoding Shenchen Pump Industry Co., Ltd., Baoding, China), then cooling to form solid medium, and can be stored at 4°C for up to a month until use.

**Table 1.** Ingredients and cost of solid medium for *Z. cucurbitae* larvae

Diet ingredients	%	Quantities	Cost ($)
Corn flour	4.33	520.0 g	0.59
Soybean meal	2.00	240.0 g	0.31
Dry yeast	6.67	800.0 g	5.46
Sucrose	4.00	480.0 g	0.59
Glucose	1.67	200.0 g	0.12
Yeast extract	0.33	40.0 g	1.88
CaCl_2_·2H_2_O	0.03	4.0 g	0.03
Agar	0.37	44.0 g	2.31
Pumpkin	6.67	800.0 g	0.45
Water	83.33	10,000.0 ml	0.00
Propionic acid	0.63	48.0 ml	1.16
Total		12,000.0 ml	12.89

The cost was calculated at the exchange ratio of 7.03 Chinese yuan per 1 US dollar ($).

For preparation of the control diets, the seeds and peel of pumpkin and cucumber were first removed, they were then diced and homogenized. Excessive liquid was removed and the diets were stored in a refrigerator at 4°C before use.

### Life Table Experiments

For egg collection, cucumber was cut into thin slices, and three or four slices were placed in a plastic petri dish in rearing cage to allow females to oviposit on them from 4:00 to 6:00 p.m. After 2 h, the slices were removed using forceps and eggs were collected for life table study. 560 newly hatched larvae (<24 h) each was placed individually into a petri dish (6 cm × 2 cm) containing a piece of diet (1 cm × 1 cm × 2 cm); 160, 200, and 200 larvae for cucumber, pumpkin, and the solid medium in 160, 200, and 200 separate petri dishes, respectively (Supp Fig. S1A [online only]). Every single larva was checked daily for exuviation and food was refreshed. The body weights and lengths of the larvae entering the next instar were measured. Mature larvae were then put in sand. On the third day, each pupa was collected and its length was measured using a high-end stereomicroscope (Olympus, SZX16) equipped with a light guide illumination system (Olympus, LG-PS2), SDF PLAPO 1× PF objective, and a digital camera (Olympus, DP72). After eclosion male and female adults were identified and placed into separate cages. The female adults were then mixed with males and examined for fecundity at the seventh day after the first eclosion of females, and eggs were collected until the 30th day after eclosion.

### Life Table Data Analyses

The development times, longevity, and fecundity of all Z. *cucurbitae* individuals, as well as mean values and standard errors, were analyzed using the age-stage two-sex life table implemented in the program TWOSEX-MSChart (Chi 2018). Population parameters, and their means, standard errors, and significant differences were calculated and analyzed by a one-factor analysis of variance (ANOVA) using SPSS (version 17.0). The means were analyzed using the Tukey–Kramer HSD test with *P* < 0.01. The results were plotted using GraphPad Prism 5.

## Results

### Age-Stage, Two-Sex Life Table of *Z. cucurbitae* Reared on Pumpkin, Cucumber, and Solid Medium

The development times of *Z. cucurbitae* at each stage are shown in Table 2. The development time of egg stage (*F* = 3.93; df = 2,396; *P* = 0.021) on the solid medium was 2.07 ± 0.022 d, which was similar to that of those reared on pumpkin (2.00 ± 0.00 d) and cucumber (2.04 ± 0.017 d). The duration of the first instar larvae (*F* = 52.85; df = 2,365; *P* < 0.001) on the solid medium was 2.43 ± 0.06 d, longer than 2.01 ± 0.01 d on pumpkin and 2.00 ± 0.00 d on cucumber. Similarly, the second larval stage (*F* = 82.99; df = 2,292; *P* < 0.001) on the solid medium was 1.82 ± 0.06 d, significantly longer than 1.08 ± 0.03 d on cucumber and 1.11 ± 0.04 d on pumpkin. The third larval stage was 3.53 ± 0.09 d on the solid medium, close to 3.65 ± 0.09 d on pumpkin but longer than 3.16 ± 0.05 d on cucumber. Thus, the total larval period of *Z. cucurbitae* reared on the artificial diet was 7.80 ± 0.09 d, significantly longer than 6.76 ± 0.08 d on pumpkin and 6.22 ± 0.05 d on cucumber.

**Table 2.** Development time of *Z. cucurbitae* reared on pumpkin, cucumber, and solid medium

Stage	Control				Solid medium		df	*F*	*P*
	Pumpkin		Cucumber						
	*N*	Mean ± SE (days)	*N*	Mean ± SE (days)	*N*	Mean ± SE (days)			
Egg	121	2.00 ± 0.00a	128	2.04 ± 0.017a	123	2.07 ± 0.022a	2,369	3.93	0.021
First instar	121	2.01 ± 0.01b	128	2.00 ± 0.00b	119	2.43 ± 0.06a	2,365	52.85	<0.001
Second instar	105	1.11 ± 0.04b	92	1.08 ± 0.03b	98	1.82 ± 0.06a	2,292	82.99	<0.001
Third instar	86	3.65 ± 0.09a	69	3.16 ± 0.05b	76	3.53 ± 0.09a	2.228	9.73	<0.001
Larval duration	86	6.76 ± 0.08b	69	6.22 ± 0.05c	76	7.80 ± 0.09a	2.228	97.27	<0.001
Pupae	75	9.03 ± 0.05b	52	8.92 ± 0.05b	65	9.34 ± 0.07a	2,189	13.46	<0.001
Preadult	75	17.73 ± 0.09b	52	17.13 ± 0.06c	65	19.11 ± 0.10a	2,189	128.58	<0.001

Means in the same row followed by the same letter are not significantly different (*P* > 0.01) using Tukey–Kramer procedure. df: degree freedom; *F*: *F*-statistical value; *P*: probability value.

The life span of pupal *Z. cucurbitae* reared on the solid medium was a little longer than on pumpkin or cucumber. The development time of *Z. cucurbitae* preadults was 19.11 d on the solid medium, about 1.4–2.0 d longer than that on pumpkin or cucumber (Table 2). However, the pupation rates (from egg to pupa) were 69.92%, 52.31%, and 60.00% on pumpkin, cucumber, and the solid medium, respectively. The emergence rates of melon fly obtained from pumpkin, cucumber, and the solid medium were 87.21%, 76.47%, 86.67%, respectively (Supp Table S1 [online only]). These results suggest that our artificial diet does not cause significantly negative effects on pupation and eclosion.

**Fig. 1.**
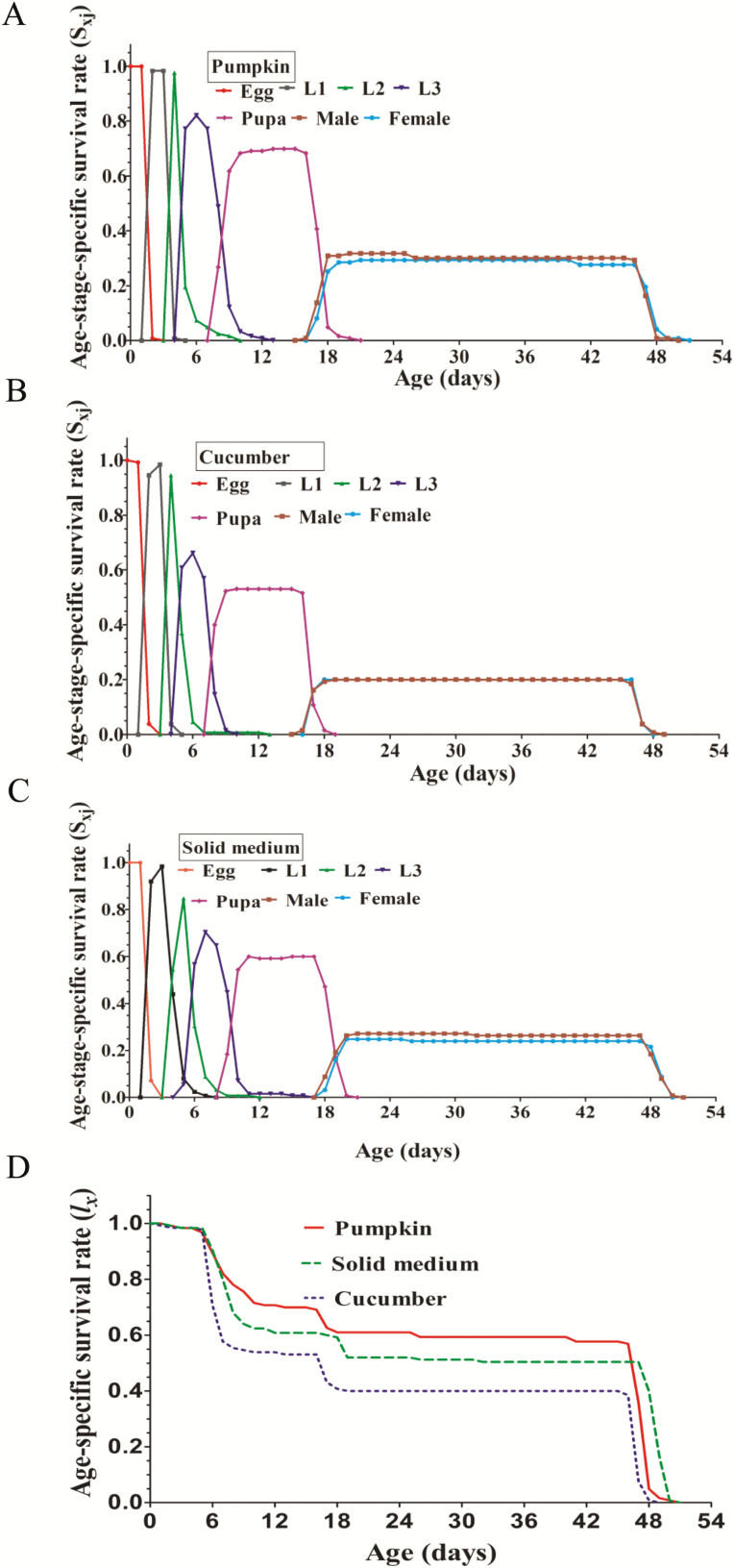
Age-stage-specific survival rate (*S*_*xj*_ and *l*_*x*_) of *Z. cucurbitae* fed pumpkin, cucumber, and solid medium. Age-Stage-Specific survival rate on pumpkin (A), cucumber (B), and solid medium (C). Age-Specific survival rates for pumpkin, cucumber, and solid medium (D).

Survival rates can be used to measure the probability that an egg will survive to certain age. The age-stage-specific survival rates of the third larvae, pupae, females, and males projected by two-sex life table were higher on the solid medium than on cucumber (Fig. 1A–C). As shown in Fig. 1D, the age-specific survival rate of *Z. cucurbitae* was higher on the solid medium than cucumber, whereas it was higher for *Z. cucurbitae* on pumpkin than the solid medium except for the days from 2 to 6. These results suggest that *Z. cucurbitae* successfully survive and reproduce with feeding pumpkin, cucumber, and the solid medium.

### Comparable Body Size and Weight of *Z. cucurbitae* Immatures of Various Stages Reared on Pumpkin, Cucumber, and Solid Medium

**Fig. 2.**
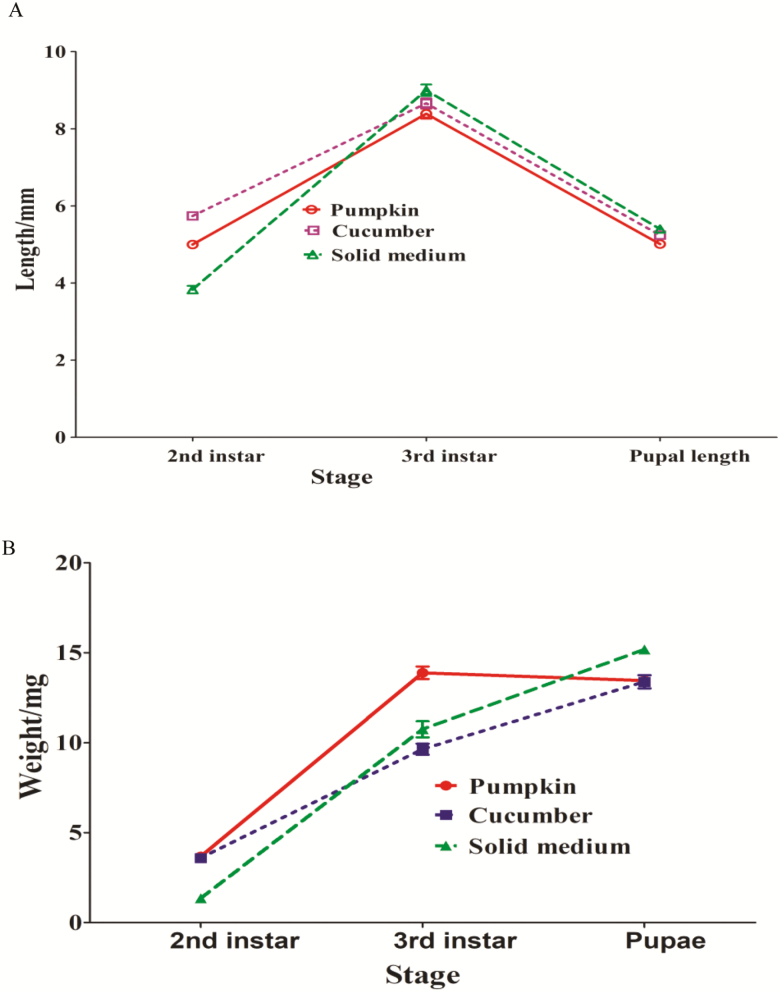
Mean body length (A) and weight (B) of *Z. cucurbitae* fed pumpkin, cucumber, and solid medium.

We compared the body sizes of immature *Z. cucurbitae* at various stages with feeding the three different diets (Fig. 2A). At the second larval stage the mean body length of *Z. cucurbitae* reared on cucumber was longer than those on the other two diets (*F* = 151.40; df = 2,240; *P* < 0.001). The mean body length of the third instar larvae on the solid medium was greater than those on the other two diets (*F* = 5.46; df = 2,329; *P* = 0.004). The mean pupal length of *Z. cucurbitae* was longer on the solid medium than on the other two diets (*F* = 31.37; df = 2,225; *P* < 0.001). However, pupal widths on the three different diets are similar, 2.38, 2.38, and 2.44 mm on pumpkin, cucumber and the solid medium, respectively.

*Zeugodacus cucurbitae* immatures on the three diets showed different weights at different stages (Fig. 2B). The mean body weight of the second larva on the solid medium was less than those on both pumpkin and cucumber. When reached to the third larval stage, the mean body weight of *Z. cucurbitae* on the solid medium was only less than that on pumpkin. Finally, *Z. cucurbitae* fed the solid medium scored the highest pupal weights (*F* = 12.43; df = 2,225; *P* < 0.001). Taken together, the body size and weight of the immatures on three different diets are comparable.

### Population Dynamics and Reproduction of *Z. cucurbitae* Reared on Pumpkin, Cucumber, and Solid Medium

**Fig. 3.**
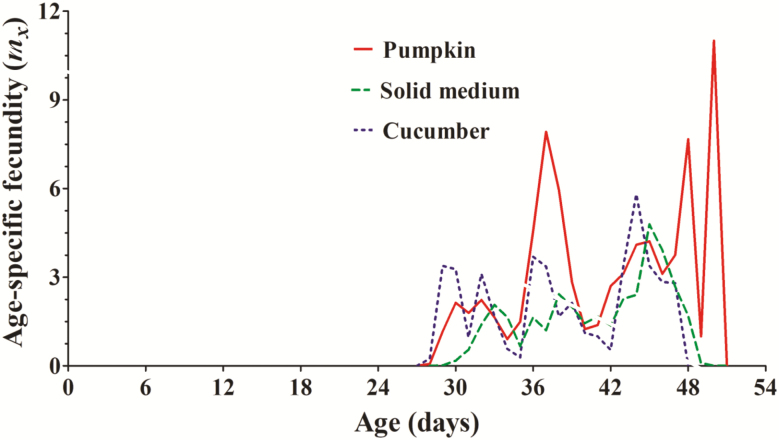
Age-specific fecundity of total population (*m*_*x*_) of *Z. cucurbitae* reared on pumpkin, cucumber, and solid medium.

Projected by two-sex life table, *Z. cucurbitae* exhibited higher net reproduction rate on the solid medium than cucumber. With regard to the intrinsic rate of increase, however, *Z. cucurbitae* reared on the solid medium was the lowest, 0.0704 per day (Table 3). The reproduction of *Z. cucurbitae* began at the age of 28 d on both pumpkin and cucumber while it started at 29 d old on the solid medium (Fig. 3). A single high peak of age-specific fecundity occurred at the age of 45 d for *Z. cucurbitae* on the solid medium (4.7937 offspring). This differed from *Z. cucurbitae* on pumpkin, which had an approximate plateau stage that occurred between four high peaks at the ages of 37, 45, 48, and 50 d (7.9178, 4.2113, 7.6667, and 11.0000 offspring each) (Fig. 3).

**Table 3.** Population parameters of *Z. cucurbitae* on pumpkin, cucumber, and solid medium

Parameters	Original			Bootstrap (mean ± SE)		
	Pumpkin	Cucumber	Solid medium	Pumpkin	Cucumber	Solid medium
*r*	0.0898	0.0751	0.0704	0.0896 ± 0.0038	0.0746 ± 0.0054	0.0700 ± 0.0043
λ	1.0940	1.0780	1.0729	1.0937 ± 0.0042	1.0775 ± 0.0058	1.0726 ± 0.0046
*T*	38.82	37.85	41.08	38.83 ± 0.1680	37.88 ± 0.3410	41.0860 ± 0.1950

*r*: intrinsic rate of increase (day^−1^); λ: finite rate of increase (day^−1^); *T*: mean generation time (days).

The reproductivity of insect can be also measured by its contribution at certain age and stage to the future population. Compared to slow increase of reproductivity from egg to pupal stages, the age-stage reproductive vales of females significantly increased to 17.2132, 19.3210, and 15.3527 at 17, 17, and 18 d on pumpkin, cucumber, and the solid medium, respectively (Supp Fig. S2A–C [online only]). The peaks of age-stage reproductive value of females occurred at the age of 36, 32, and 29 d and were 60.6710, 47.0803, and 40.7726 on pumpkin, cucumber, and the solid medium, respectively. However, the reproductivity females grew from artificial diet decreased at the lowest speed compared to those from the two controls.

## Discussion

In this study, we used age-stage, two-sex life table method to examine the fitness parameters of *Z. cucurbitae* with larvae reared on a novel solid medium that has been used for more than 2 yr in our laboratory (Supp Fig. S1B [online only]). The results demonstrate that our solid medium is comparable with natural host plants pumpkin and cucumber, making it an excellent artificial diet of *Z. cucurbitae* for laboratory research and possible mass rearing.

The growth parameters, such as developmental time, body size, and survival rate, can be used to evaluate artificial diets. In our study, the total preadult development duration of *Z. cucurbitae* is 19.11 d on the artificial diet, and 17.73 d on pumpkin. Both of them are in the range of 15–19 d reported in previous studies on various natural host plants and artificial diets (Vayssières et al. 2008, Huang and Chi 2012, Mir et al. 2014, Jaleel et al. 2018). For example, Vayssières et al. (2008) reported that the total preadult development duration of *Z. cucurbitae* on cucumber was 17.2 d, which is in agreement with our results, 17.1 d on cucumber; Jaleel et al. (2018) showed that the preadult development takes 16.3 d. The differences of development time on different diets are probably due to different nutrient compositions and ratios. With regard to the body size, *Z. cucurbitae* reared on the solid medium reaches to similar level to those reared on the other two nature hosts (Supp Fig. S2 [online only]). In addition, the age-specific survival rate of *Z. cucurbitae* is higher on the solid medium than cucumber although lower than pumpkin.

The reproductive capability of insect is also an important for an artificial diet. In our study, the intrinsic rates of increase were 0.0898, 0.07 51, and 0.0704 per day for flies reared on pumpkin, cucumber, and our solid medium, respectively. The intrinsic rates of increase of *Z. cucurbitae* on the artificial diet are the lowest. The reason might be partially due to its longer mean generation time on the artificial diet than the other two diets (Table 3). The intrinsic rates in our study are lower than those from a previous study, in which the intrinsic rates of 0.1412, 0.1446, and 0.0688 per day have been reported for *Z. cucurbitae* reared on pumpkin, cucumber, and carrot medium, respectively (Huang and Chi 2012). Jaleel et al. (2018) reported an intrinsic rate of 0.109 per day for *Z. cucurbitae* reared on a semiartificial diet. The difference may primarily result from different nutrients and culture conditions. Nevertheless, the reproductive parameters of *Z. cucurbitae* on our artificial diet are almost comparable to that of two natural food. Taken together, our results suggest the growth and reproduction of *Z. cucurbitae* on our artificial diet is normal or almost normal.

There are mainly two artificial diets for *Z. cucurbitae* larvae. The first one has been developed for mass rearing tephritid fruit flies; however, it requires substantial cost, storage room, and labor (Tanaka et al. 1969). In contrast, as all ingredients in our artificial diet can be ingested by *Z. cucurbitae* larvae, there will be rarely diet leftover by adjusting the amount of artificial diet. It is easy to be cleaned even if there is a small amount of used diet left in petri dish or tray, which is environment- and labor-friendly. Since there is no bulky agent such as mill feed, the requirement for environment-controlled storage space is significantly reduced. Compared with the liquid diet (Chang et al. 2004), our solid medium does not require special care for earlier larval melon fly and sponge cloth that needs room to store and to be cleaned before use. Our artificial diet is also easy to make and be aliquoted, and can be stored for 2 wk to 1 mo without affecting larval growth of *Z. cucurbitae* (unpublished data). Additional reagents can be accurately added into it according to research requirement. Particularly, the cost of our artificial diet is very low, only $12.89 per 12 liters, approximately $1 per 1 liter. However, the liquid diet was $33.06 per 1.5 liters (Chang et al. 2004) and the cost of Tanana’s recipe is similar to the liquid diet (Tanaka et al. 1969).

In conclusion, we used the age-stage, two-sex life table method to investigate whether our novel solid medium was suitable for rearing of *Z. cucurbitae* larvae with comparison to the two natural hosts of pumpkin and cucumber. The data demonstrate that our artificial diet shows apparent advantages in saving cost, labor, storage space besides normal or almost normal development and growth of *Z. cucurbitae*, therefore will be beneficial for its research in laboratory and possible practical applications, such as SIT and genetic control.

## Supplementary Material

ieaa058_suppl_Supplemenatary_Table_S1

ieaa058_suppl_Supplemenatary_Figures
