# Supplementary material for: A Novel Solid Artificial Diet for Zeugodacus cucurbitae (Diptera: Tephritidae) Larvae With Fitness Parameters Assessed by Two-Sex Life Table
Source: J Insect Sci. 2020 Aug 18;20(4):21. doi: 10.1093/jisesa/ieaa058 (PMC7433769; doi:10.1093/jisesa/ieaa058)
Supplement: ieaa058_suppl_Supplemenatary_Table_S1 [file ieaa058_suppl_supplemenatary_table_s1.docx]

**Table S1.** Survival rate of *Z. cucurbitae* reared on pumpkin, cucumber and solid medium.

| Survival rate | Control | | Solid medium |
| --- | --- | --- | --- |
|  | Pumpkin | Cucumber |  |
| Pupation rate (%) | 69.92 | 52.31 | 60.00 |
| Emergence rate (%) | 87.21 | 76.47 | 86.67 |
