# Supplementary figures and images for: A Novel Solid Artificial Diet for Zeugodacus cucurbitae (Diptera: Tephritidae) Larvae With Fitness Parameters Assessed by Two-Sex Life Table
Source: J Insect Sci. 2020 Aug 18;20(4):21. doi: 10.1093/jisesa/ieaa058 (PMC7433769; doi:10.1093/jisesa/ieaa058)

A

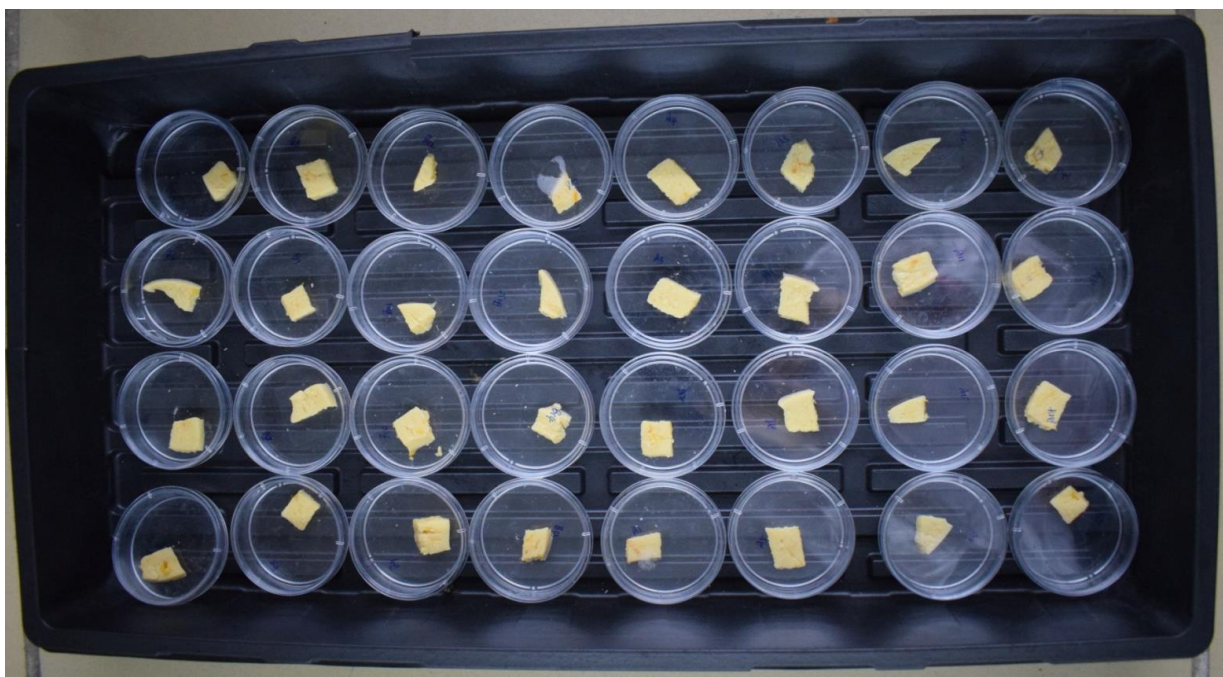

B

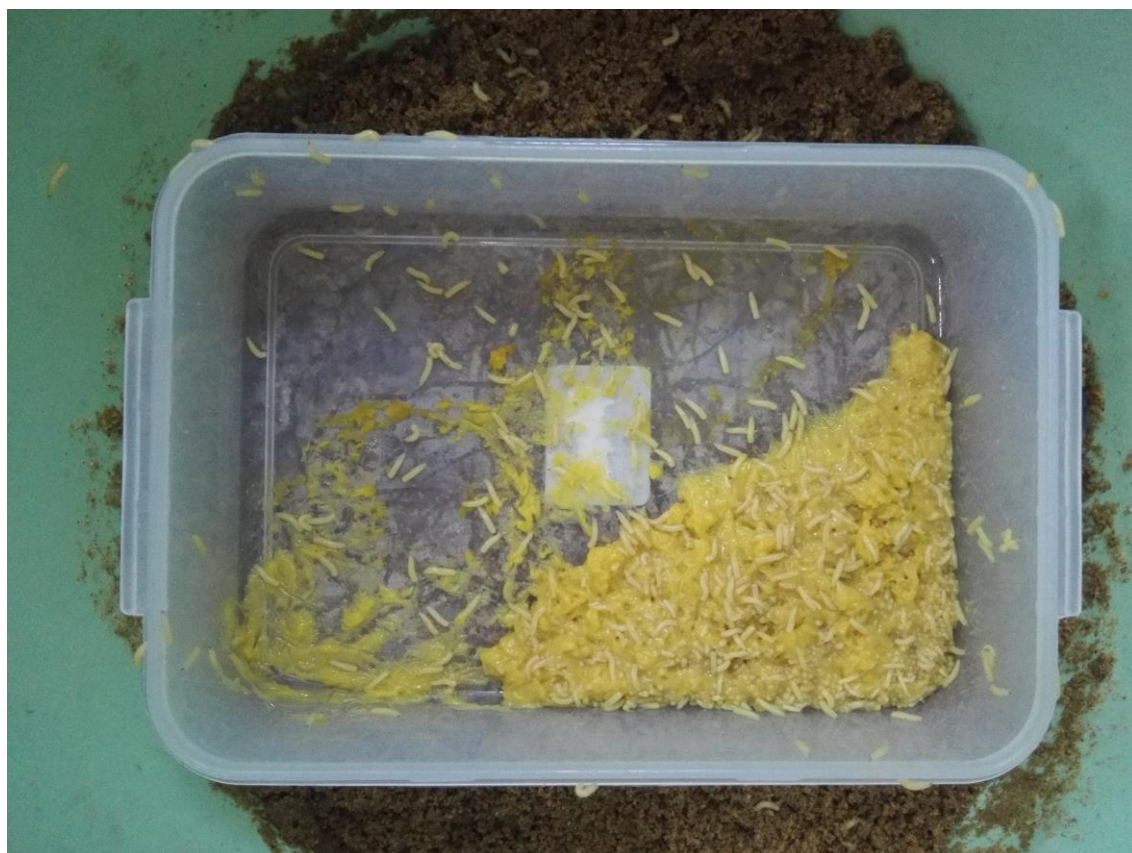

**Figure S1**

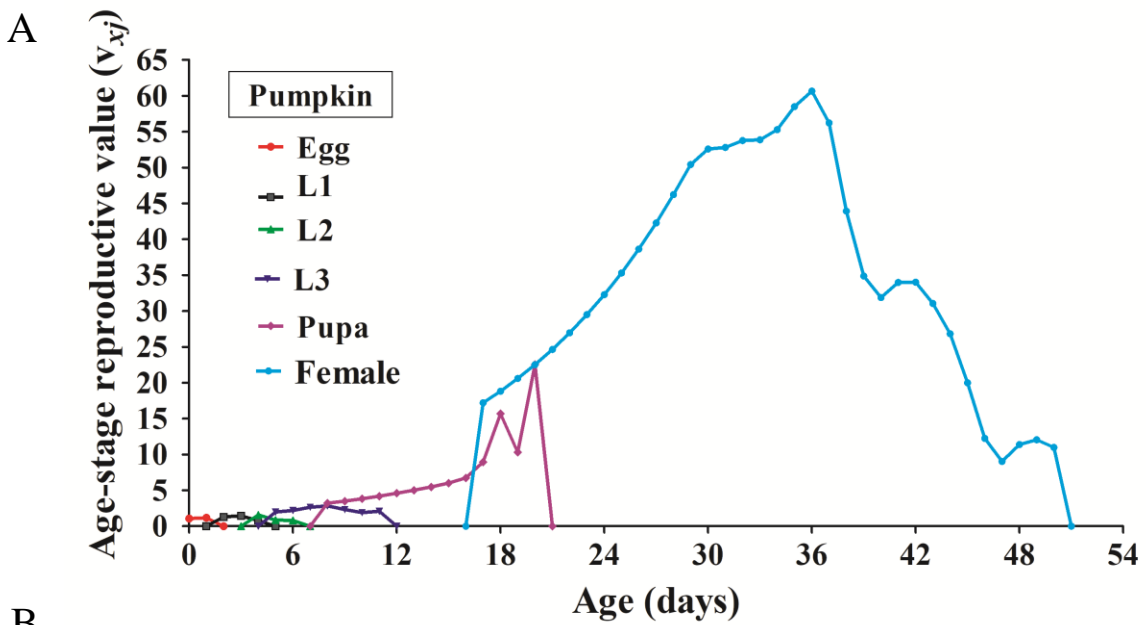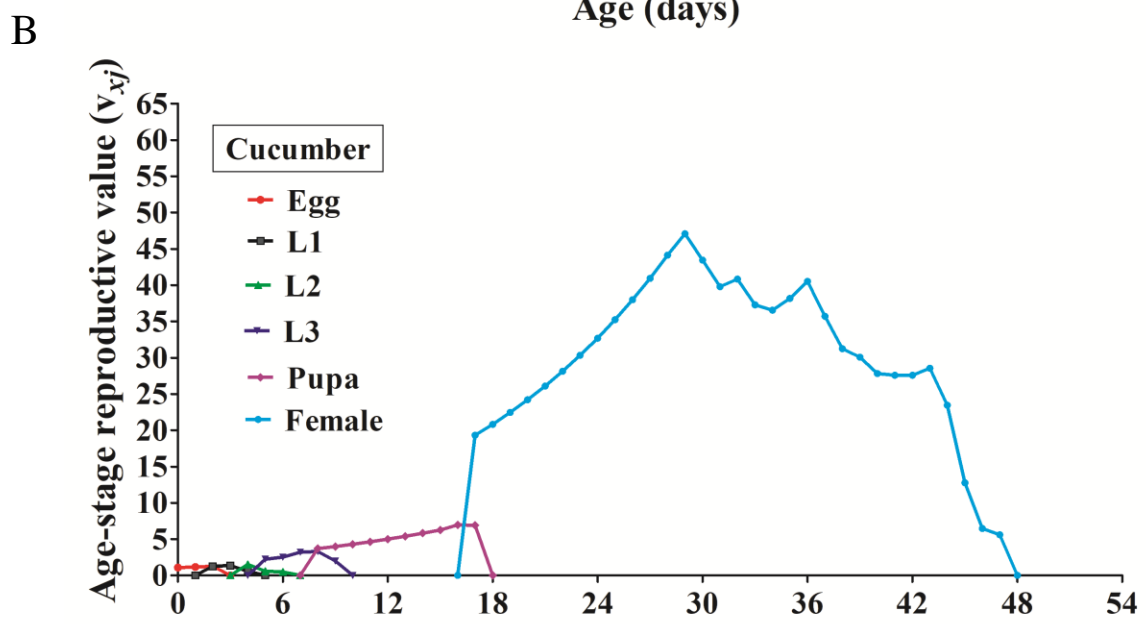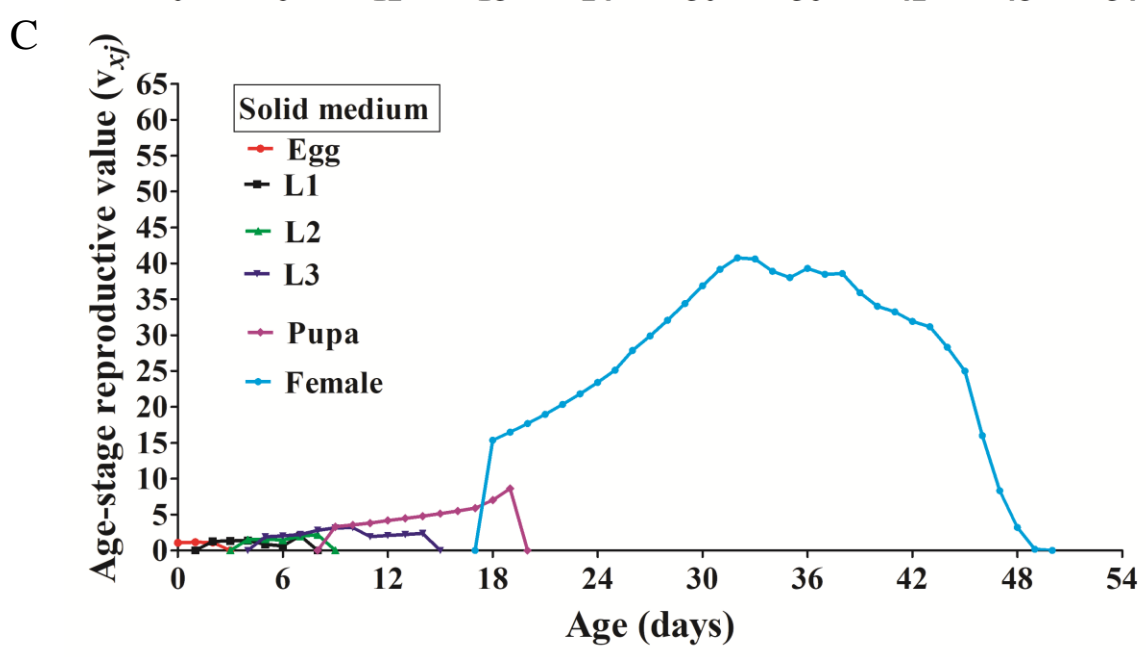

**Figure S2**

Supplement: ieaa058_suppl_Supplemenatary_Figures [file ieaa058_suppl_supplemenatary_figures.pdf]
